# 

**Published:** 1858-10

**Authors:** 


VOL. I.] PUBLISHED MONTHLY. [No. 10.

### THE CHICAGO

### MEDICAL JOURNAL

EDITED BY

N. S. DAVIS, M.D.,

Professor of Principles and Practice of Medicine in Rush Medical College,

AND

W. H. BYFORD, M.D.,

Professor of Obstetrics and Diseases of Women and Children in Rush Medical College.

OCTOBER, 1858.

JAS. BARNET, BOOK & JOB PRINTER, 189 LAKE ST., CHICAGO, ILL.

AT TWO DOLLARS PER ANNUM, IN ADVANCE.

